# Glutaminase C Regulates Microglial Activation and Pro-inflammatory Exosome Release: Relevance to the Pathogenesis of Alzheimer’s Disease

**DOI:** 10.3389/fncel.2019.00264

**Published:** 2019-06-28

**Authors:** Ge Gao, Shu Zhao, Xiaohuan Xia, Chunhong Li, Congcong Li, Chenhui Ji, Shiyang Sheng, Yalin Tang, Jie Zhu, Yi Wang, Yunlong Huang, Jialin C. Zheng

**Affiliations:** ^1^Center for Translational Neurodegeneration and Regenerative Therapy, Shanghai Tenth People’s Hospital Affiliated to Tongji University School of Medicine, Shanghai, China; ^2^Department of Pharmacology and Experimental Neuroscience, University of Nebraska Medical Center, Omaha, NE, United States; ^3^Collaborative Innovation Center for Brain Science, Tongji University, Shanghai, China

**Keywords:** Alzheimer’s disease, glutaminase C, microglia activation, brain inflammation, glutaminase inhibitor, exosome

## Abstract

Microglial activation is a key pathogenic process at the onset of Alzheimer’s disease (AD). Identifying regulators of microglial activation bears great potential in elucidating causes and mechanisms of AD and determining candidates for early intervention. Previous studies demonstrate abnormal elevation of glutaminase C (GAC) in HIV-infected or immune-activated microglia. However, whether GAC elevation causes microglial activation remains unknown. In this study, we found heightened expression levels of GAC in early AD mouse brain tissues compared with those in control littermates. Investigations on an *in vitro* neuroinflammation model revealed that GAC is increased in primary mouse microglia following pro-inflammatory stimulation. To model GAC elevation we overexpressed GAC by plasmid transfection and observed that GAC-overexpression shift the microglial phenotype to a pro-inflammatory state. Treatment with BPTES, a glutaminase inhibitor, reversed LPS-induced microglial activation and inflammation. Furthermore, we discovered that GAC overexpression in mouse microglia increased exosome release and changed exosome content, which includes specific packaging of pro-inflammatory miRNAs that activate microglia. Together, our results demonstrate a causal effect of GAC elevation on microglial activation and exosome release, both of which promote the establishment of a pro-inflammatory microenvironment. Therefore, GAC may have important relevance to the pathogenesis of AD.

## Introduction

Alzheimer’s disease (AD) is currently the most common neurodegenerative disease worldwide, with an incidence of approximate 6% among population over 65, and around 20% over 80 (Danborg et al., 2014; Reitz and Mayeux, 2014). It is the number one cause for dementia in the aged population, imposing significant personal, societal, and economic tolls (WHO 2018 Annual Report). Chronic inflammation and neuronal damage are key processes of AD (Sardi et al., 2011). Microglia are the resident immune cells in the CNS and form the first line of defense during brain injury or disease (Block and Hong, 2005; Glass et al., 2010). The activation of microglia plays a central role in neuroinflammation during AD. Pathogenesis of AD is known to involve accumulation of amyloid peptides (Aβ) that leads to microglial activation and release of pro-inflammatory mediators such as cytokines, reactive oxygen species, and toxic chemicals. These mediators include several neurotoxic secretory products that may ultimately cause severe neurotoxicity and loss of synaptic connections (Tan et al., 1999; Sastre et al., 2006).

Our previous studies demonstrated that the expression of GLS1 is up-regulated in HIV-1-infected macrophages and microglia, which causes neurotoxicity and serves as a key pathogenic process in HIV-1-associated neurocognitive disorders (Huang et al., 2011). GLS1 is the enzyme catalyzing the hydrolysis of glutamine to produce glutamate in the CNS (Curthoys and Watford, 1995). It has two variants due to alternative splicing, including KGA and GAC (Elgadi et al., 1999; Porter et al., 2002). GAC is the variant that is elevated in HIV-1-infected macrophages and microglia (Huang et al., 2011). Interestingly, transgenic mice with GAC overexpression in neural stem/progenitor cells (NPC) and neural cells-derived from NPC (Nestin-GAC mice) exhibited microglial activation, brain inflammation, and memory deficits (Wang et al., 2017) similar to those key pathogenic processes as seen in AD. However, whether GAC is altered in AD-related neuroinflammation and whether GAC alteration directly instigates microglial activation and brain inflammation remain unknown.

In this study, we found that the expression levels of GAC, but not KGA, were elevated in AD transgenic mouse brain tissues compared with those in control littermates. Notably, GAC elevation was in concurrence with increased expression levels of pro-inflammatory markers. GAC overexpression in resting microglia polarized microglia into an active and pro-inflammatory state. Furthermore, GAC overexpression increased the release of exosomes that contain specific packaging of pro-inflammatory miRNAs, which may promote a pro-inflammatory circumstance for microglial activation.

## Materials and Methods

### Mice and Microglia Culture

APP/PS1 mice and C57 mice (purchased from Shanghai Model Organisms Center) are housed and bred in the Comparative Medicine animal facilities of Tongji University School of Medicine (TUSM). All procedures were conducted according to protocols approved by the Institutional Animal Care and Use Committee of TUSM. Mouse genotype was validated by PCR. Mouse primary microglia was isolated from whole brains of C57 mice at postnatal day 1 (P1). Mouse brains were dissected out after removing peripheral blood vessels and washed twice with HBSS. Next, mouse brains were digested at 37°C for 30 min in 0.25% trypsin solution supplemented with 0.05% DNase I. Digestion was stopped by FBS (Invitrogen). The tissue sediment was centrifuged at 1500 rpm for 5 min at 4°C and washed twice with HBSS. After trituration, cells were plated and cultured in DMEM with 10 ng/mL GM-CSF and 10% FBS, 50 U penicillin and 50 mg/mL streptomycin at 37°C. Culture dishes or plates were coated with 100 μg/mL Poly-D-Lysine (Sigma) and 5 μg/mL Fibronectin (Sigma). The culture medium was replaced every 3 days. Cells were subjected to three passages for purification purpose. Microglia purity was confirmed by immunostaining with antibodies against Iba1 (cat# 019-19741, WAKO).

### Plasmid Transfection for GAC Overexpression

Plasmids expressing human KGA and GAC were commercially purchased (Jikai, Inc.). Cultured mouse microglia were transfected by plasmids with KGA or GAC expression with Lipofectmine2000 (Life Technologies, Inc.) according to the manufacture’s instruction for 48 h before collected for further analyses.

### Protein Extraction and Western Blot

Mice were euthanized and brains were removed and homogenized by a homogenizer in the M-PER Protein Extraction Buffer (Pierce) containing a protease inhibitor cocktail (Sigma). Protein concentrations were determined with a BCA Protein Assay Kit (Pierce). Proteins (5−10 μg) from tissue lysates or proteins (20–30 μg) from cell lysates were separated by sodium dodecyl sulfate polyacrylamide gel electrophoresis (SDS-PAGE) and electrophoretic transferred to polyvinylidene fluoride membranes (Millipore and Bio-Rad). Membranes were incubated with primary antibodies for CD86 (rabbit, cat#ab86392, Abcam, 1:1000), GLS1 (rabbit, cat#ab156876, Abcam, 1:1000), CD206 (mouse, cat#AF2535, R&D Systems, 1:500), neuron-specific Class III β-tubulin (Tuj1) (rabbit, cat#T2200, Sigma-Aldrich, 1:1000), Flotillin-1 (mouse, cat#610821, BD Biosciences, 1:1000), or β-actin (Sigma-Aldrich) overnight at 4°C followed by a horseradish peroxidase-linked secondary anti-rabbit or anti-mouse antibody (Cell Signaling Technologies, 1:10,000) incubation. Antigen-antibody complexes were visualized by Pierce ECL Western Blotting Substrate (Thermo Fisher Scientific, Waltham, MA, United States). For data quantification, films were scanned with a CanonScan 9950F scanner; the acquired images were analyzed using the free public domain NIH ImageJ program^1^.

### Enzyme-Linked Immunosorbent Assay (ELISA)

Serum of cultured primary mouse microglia with/without GAC overexpression was collected and pro-inflammatory cytokine TNF-α was measured with commercially available ELISA kits (cat# 50349-MNAE, Sino Biological) according to manufacturer’s protocols.

### Immunochemistry

Immunochemistry was done as previously described (Ma et al., 2019). Briefly, cells or tissue sections were fixed in 4% paraformaldehyde (Sigma) for 15 min at room temperature (RT), washed 3 times with PBS (Thermo Fisher Scientific), and incubated with permeabilizing and blocking buffer containing 5% goat serum (Vector Laboratories) and 0.2% Triton X-100 (Bio-Rad) in PBS for 1 h at RT. Fixed cells or tissue sections were incubated with primary antibody for Iba1 (goat, cat# ab5076, Abcam, 1:500), GLS1 (rabbit, cat# ab156876, Abcam, 1:2000), GFAP (rabbit, cat# Z0334, DAKO, 1:500), or neuron-specific Class III β-tubulin (Tuj1, rabbit, cat# T2200, Sigma-Aldrich, 1:500) overnight at 4°C. The next day, cells or tissue sections were washed with PBS and incubated with secondary antibodies (Molecular Probes) for 1 h at RT. Cells were counterstained with DAPI (Sigma-Aldrich). Images were taken using a Nikon Eclipse E800 microscope equipped with a digital imaging system and imported into Image-Pro Plus, version 7.0 (Media Cybernetics) for quantification. 600–1,000 immunostained cells from 15 randomly picked fields per group were counted.

### RNA Isolation and qPCR Analysis

Total RNA was isolated by Purification Kit (Fermentas) with DNase I digestion (Qiagen) to remove genomic DNA. Messenger RNA – derived cDNA was generated using Oligo-dT priming with Transcriptor First Strand cDNA Synthesis Kit (Roche). RNase inhibitor was used to prevent degradation. Amplification was performed using SYBR Green PCR Master Mix (Applied Biosystems) and specific primer sets (Supplementary Table S1). GAPDH was used for the normalization of all mRNA expression levels.

### Analyses of Glutamate by Amplex Red Glutamic Acid/Glutamate Oxidase Assay Kit

Intracellular and extracellular glutamate levels in microglia culture were determined by Amplex Red Glutamic Acid/ Glutamate Oxidase Assay Kit (Invitrogen, A12221) based on the manufacture’s instruction. For intracellular glutamate assay, cellular protein lysates were diluted to the same protein concentration before entering the assay. For extracellular glutamate assay, culture media was changed to a phenol red-free media 24 h before the assay, and same volumes of supernatant were entered into the assay.

### Isolation of Exosomes

Exosomes were isolated from the serum-free microglia culture as previously described (Wu et al., 2018). Gradient ultracentrifugation was utilized for exosomes isolation. Briefly, cells were planted on poly-L-Ornithine/laminin-coated 10 cm dish and cultured in for 12 h. First, supernatants were centrifuged at 300 × *g* for 10 min to remove free cells, then at 3000 × *g* for 20 min to remove debris, and 10,000 × *g* for 30 min to remove organelles. Exosomes were collected through ultracentrifugation at 100,000 × *g* for 2 h. All centrifugation steps were performed at 4°C.

### Nanoparticle Tracking Analysis (NTA)

The size and number of exosomes were determined as previously described (Wu et al., 2018). Briefly, microglial cells were planted on poly-L-Ornithine/laminin-coated 10 cm dish and cultured. The supernatants of cultured cells were collected after 12 h, and the collected exosomes were resuspended in 150 μl PBS and diluted at 1:100 in PBS. One milliliter solution was used for NanoSight analysis. NTA was done on NanoSight NS300 system (Malvern Instruments, United Kingdom) with a sCMOS camera. The conditions of the measurements were set at 25°C, 1 cP viscosity, 25 s per capture frame and 60 s for measurement time. Three individual measurements were performed for the measurement of sizes and concentrations of exosomes.

### Statistical Analyses

Data from two groups were evaluated statistically by two-tailed, paired or unpaired student *t*-test. Data were shown as mean ± SD, and significance was determined as *P* < 0.05.

## Results

### GAC Expression Is Elevated in Early AD Mouse Brain Tissues

**FIGURE 1:**
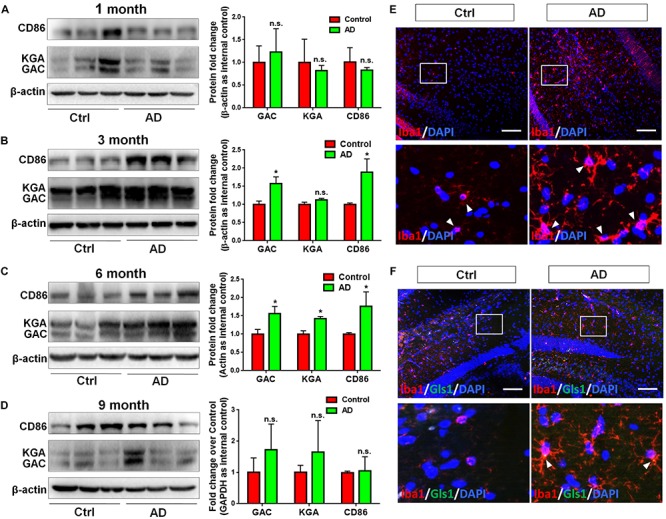
Upregulation of GAC in early stage AD mouse brain tissues. **(A–D)** Brains of 1, 3, 6, and 9 months APP/PS1 mice and their control littermates were removed and homogenized for protein analyses. Representative Western blots were shown and CD86, KGA, and GAC protein expression levels in 1 month APP/PS1 and control mouse brains **(A)**, 3 month APP/PS1 and control mouse brains **(B)**, 6 month APP/PS1 and control mouse brains **(C)**, 9 month APP/PS1 and control mouse brains **(D)** were normalized to β-actin and presented as fold change compared with those in control mouse brains. Error bars denote s.d. from triplicate measurements. ^*^P < 0.05, by two-tailed *t*-test (*n* = 3). **(E,F)** Brains of 3 and 6 month APP/PS1 mice and their control littermates were removed after intracranial perfusion and prepared for immunofluorescence staining. Representative pictures of Iba1 expression in 3 month APP/PS1 and control mouse brain **(E)**, GLS1 and Iba1 expressions in 6 month APP/PS1 and control mouse brain **(F)** were shown. Images at the lower panels were high-magnification images of the corresponding small box area from the upper panels. Scale bar: 10 μm.

In order to determine whether GLS1 expression was altered in the pathogenic process of AD, we investigated the protein expression levels of both KGA and GAC in APP/PS1 mouse brains. We found that the protein expression levels of KGA, GAC, and the pro-inflammatory marker CD86 were not changed in 1 month (1 M) APP/PS1 mouse brain compared with those in 1 M control mouse brains (Figure 1A). Interestingly, in 3 months (3 M) mouse brain, the expression levels of GAC and CD86 were higher in APP/PS1 mice than those in control littermates. In contrast, KGA expression levels did not show significant difference between the two groups (Figure 1B). In 6 months (6 M) mouse brain, protein expression levels of KGA, GAC, and CD86 were higher in APP/PS1 mice than those in control littermates (Figure 1C). However, at 9 months (9 M), the protein expression levels of KGA, GAC, or CD86 no longer displayed significant difference compared with those in control littermates (Figure 1D). The increase of GAC at 3 M was in concurrence with an increase of microglial activation in 3 M AD mouse brain as evidenced by more Iba1^+^ activated microglia in 3 M AD mouse hippocampus, compared to healthy controls (Figure 1E). More importantly, GLS1 co-localized with Iba1 in 3 M AD mouse hippocampus (Figure 1F). The co-localization of GLS1 and Iba1 could also be observed in 18 M AD mouse hippocampus (Supplementary Figure S1). Together, these data demonstrate an elevation of GLS1 isoforms at early stages of AD (3–6 M in APP/PS1 mouse) mouse brains in concurrence with the activation of microglia.

### GAC Is Specifically Up-Regulated in Pro-inflammatory Microglia

**FIGURE 2:**
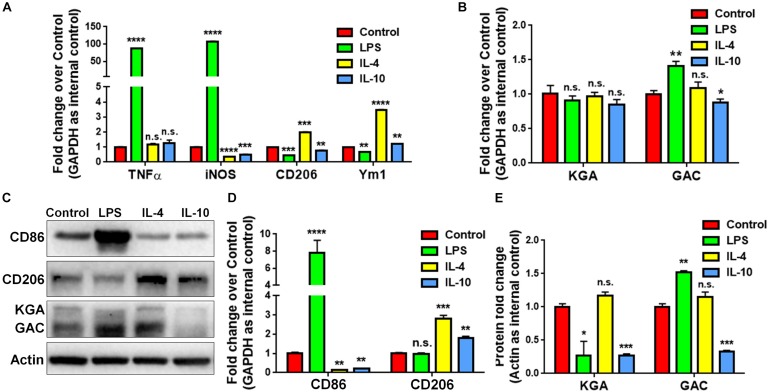
Upregulation of GAC in pro-inflammatory mouse microglia. **(A–E)** Primary mouse microglia were treated with LPS (100 ng/mL), IL-4 (50 ng/mL), and IL-10 (50 ng/mL). Total RNA was collected after the treatment for 6 h **(A,B)**. Protein lysates were collected after 24 h **(C–E)**. mRNA levels of *TNF-α*, *iNOS*, *CD206*, and *Ym1* **(A)** and *KGA* and *GAC* **(B)** were determined by qPCR analyses. Data were normalized to *GAPDH* and presented as fold change compared to control microglia. Error bars denote s.d. from triplicate measurements. Representative immunoblots of CD86, CD206, KGA, and GAC protein levels **(C)** were shown and CD86, CD206 **(D)**, KGA, and GAC **(E)** protein expression levels were normalized to β-actin and presented as fold change compared to control microglia. Error bars denote s.d. from triplicate measurements. ^*^P < 0.05, ^∗∗^*P* < 0.01, ^∗∗∗^*P* < 0.001, ^****^P < 0.0001, by two-tailed *t*-test (*n* = 3). Experiments were carried out three times in triplicates with 5–7 P1 mice per group.

To study whether GAC expression was altered under pro-inflammatory and anti-inflammatory states, we treated cultured mouse microglia with LPS (100 ng/mL), IL-4 (50 ng/mL), or IL-10 (50 ng/mL) to induce the pro-inflammatory, anti-inflammatory, and de-activated phenotypes of microglia, respectively. The enrichment of mouse microglia was validated by co-immunostaining of Iba1 with glia marker GFAP and neuronal marker Tuj1. Almost all cells expressed immunoreactivity corresponding to Iba1 but not GFAP or Tuj1 (Supplementary Figure S2). Total RNA was collected at 6 h and protein lysates were collected at 24 h after LPS, IL-4, or IL-10 treatment. The mRNA levels of pro-inflammatory markers, tumor necrosis factor–α (*TNF-α*) and inducible nitric oxide synthase (*iNOS*) were significantly elevated after LPS treatment, but either remained the same (*TNF-α*) or decreased (*iNOS*) after IL-4 or IL-10 treatment (Figure 2A). Anti-inflammatory markers *CD206* and *Ym1* were decreased in LPS-treated microglia but increased in IL-4-treated microglia (Figure 2A). Importantly, the mRNA levels of *GAC*, but not those of *KGA*, were significantly elevated in LPS-treated microglia and decreased in IL-10-treated microglia (Figure 2B). In accordance with mRNA results, protein analyses revealed increase of GAC (but not KGA) and CD86 in LPS-treated microglia (Figures 2C,D). IL-10 treatment increased CD206 protein expression, but decreased CD86, KGA, and GAC protein expressions levels in microglia (Figures 2C–E). These data demonstrate a strong association between GAC expression and the pro-inflammatory phenotype of microglia, suggesting that GAC may involve in the activation of microglia.

### GAC Overexpression Induces Microglial Activation

**FIGURE 3:**
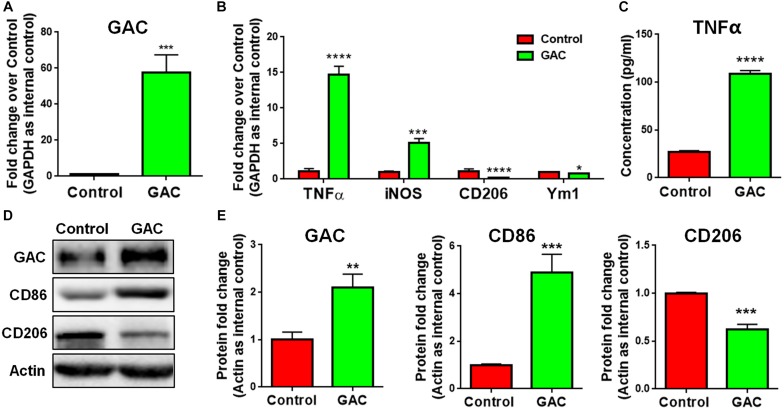
GAC overexpression induces microglial activation. **(A–E)** Primary mouse microglia were transfected with GAC overexpressing plasmid for 48 h. Total RNA and protein lysates were collected. mRNA levels of *GAC* **(A)**, *TNF-α*, *iNOS*, *CD206*, and *Ym1* **(B)** were determined by qPCR analyses. Data were normalized to *GAPDH* and presented as fold change compared to control microglia. Error bars denote s.d. from triplicate measurements. Concentrations of released TNF-α from microglia mere determined by ELISA **(C)**. Error bars denote s.d. from triplicate measurements. Representative immunoblots of GAC, CD86, and CD206 **(D)** were shown along with quantifications of GAC, CD86, and CD206 protein expression levels **(E)**. Data were normalized to β-actin and presented as fold change compared to control microglia. Error bars denote s.d. from triplicate measurements. ^*^P < 0.05, ^∗∗^*P* < 0.01, ^∗∗∗^*P* < 0.001, ^****^P < 0.0001, by two-tailed *t*-test (*n* = 3). Experiments were carried out three times in triplicates with 5–7 P1 mice per group for *in vitro* perturbation.

To test our premise that GAC is sufficient to induce microglial activation, we overexpressed GAC in primary mouse microglia by plasmid transfection. We first confirmed that GAC was indeed overexpressed in cultured cells by qPCR (60-fold increase, Figure 3A) and Western blots (2-fold increase, Figures 3D,E). Next, we examined the expression of pro-inflammatory and anti-inflammatory genes and found that the mRNA levels of pro-inflammatory genes *TNF-α* and *iNOS* were elevated in GAC-overexpressed microglia (Figure 3B). Protein analysis revealed an increased release of TNF-α and increased expression of CD86 in GAC-overexpressed microglia (Figures 3C–E). On contrary to the elevation of pro-inflammatory molecules, mRNA levels of anti-inflammatory molecules *CD206* and *Ym1* were found to be decreased after GAC overexpression (Figure 3B). Consistent with the mRNA decrease, protein analysis revealed a decrease of CD206 protein levels in microglia after GAC overexpression (Figures 3D,E). Interestingly, KGA overexpression in microglia did not activate microglia or cause neuroinflammation (Supplementary Figure S3). Therefore, these data demonstrate that elevated levels of GAC, but not KGA, are sufficient to activate microglia and induce microglia into a pro-inflammatory phenotype.

### GAC Mediates LPS-Induced Microglial Activation

**FIGURE 4:**
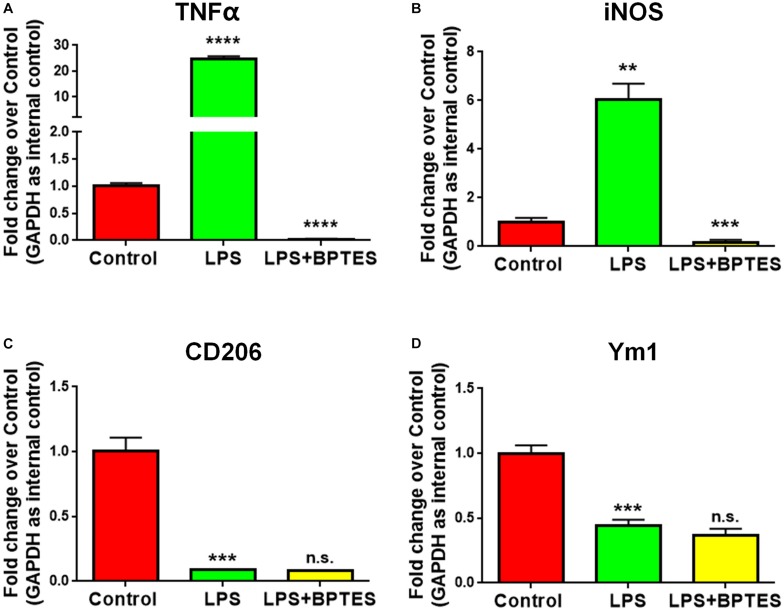
Treatment with glutaminase inhibitor reverses LPS-induced microglial activation. **(A–D)** Mouse microglia were treated with or without BPTES (10 μM) for 1 h before being exposed to LPS (50 ng/mL) for 6 h. Total RNA was collected for qPCR analyses. mRNA levels of *TNF-α* **(A)**, *iNOS* **(B)**, *CD206* **(C)**, *Ym1* **(D)** were determined by qPCR analyses. Data were normalized to *GAPDH* and presented as fold change compared to control microglia. Error bars denote s.d. from triplicate measurements. ^∗∗^*P* < 0.01, ^∗∗∗^*P* < 0.001, ^****^P < 0.0001, as compared to control microglia, by two-tailed *t*-test (*n* = 3); Experiments were carried out three times in triplicates with 5–7 P1 mice per group.

To further determine whether GAC activity is critical for pro-inflammatory transformation of microglia during LPS activation, we used BPTES, a glutaminase inhibitor, to suppress GAC activity when microglia were treated with LPS. BPTES (10 μM) was added to microglia cultures for 1 h before treatment with LPS (50 ng/mL) for 6 h. As expected, LPS treatment dramatically increased the mRNA levels of pro-inflammatory markers *TNF-α* and *iNOS*, and BPTES abolished such increases (Figures 4A,B). In comparison, LPS treatment significantly decreased the mRNA levels of anti-inflammatory markers *CD206* and *Ym1*. However, BPTES failed to reverse such decreases (Figures 4C,D). These results demonstrate that LPS-induced microglial activation through elevation of GAC activities.

### GAC-Induced Microglial Activation Is Not Through Autocrine Secretion of Glutamate

**FIGURE 5:**
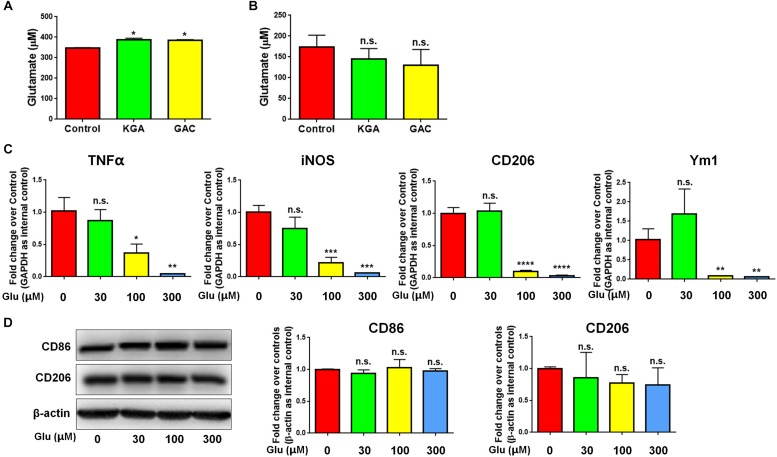
GAC overexpression induces neuroinflammation in a glutamate-independent manner. **(A,B)** Intracellular glutamate levels **(A)** and extracellular glutamate levels **(B)** in control, KGA-, and GAC-overexpressed microglia cultures were determined through the Glutamate Assay Kit. **(C)** mRNA levels of *TNF-α*, *iNOS*, *CD206*, and *Ym1* in microglia treated with different doses of glutamate were determined by qPCR analyses. Data were normalized to *GAPDH* and presented as fold change compared to control microglia. **(D)** Representative blots and quantification of protein levels of CD86 and CD206 in microglia treated with different doses of glutamate. Western blot data were normalized to β-actin and presented as fold change compared to control microglia. Error bars denote s.d. from triplicate measurements. ^*^P < 0.05, ^∗∗^*P* < 0.01, ^∗∗∗^*P* < 0.001, ^****^P < 0.0001, as compared to control microglia, by two-tailed *t*-test (*n* = 3); Experiments were carried out three times in triplicates with 5–7 P1 mice per group.

To identify whether GAC overexpression induced microglial activation via an autocrine secretion of glutamate, we first measured both intracellular and extracellular glutamate levels in microglial cultures after KGA or GAC overexpression. Intracellular glutamate was found increased in KGA- and GAC-overexpressed microglia culture (Figure 5A), but extracellular glutamate was not significantly changed in microglia culture with either KGA or GAC overexpression, indicating GAC did not regulate microglial activation through elevating extracellular glutamate (Figure 5B). To further exclude the effect of extracellular glutamate, we directly added different doses of glutamate to microglia cultures for 24 h and found that extra glutamate did not promote microglial activation but instead decreased mRNA levels of *TNF-α*, *iNOS*, *CD206*, and *Ym1* at 100 or 300 μM concentration (Figure 5C). Protein analysis revealed no change of CD86 or CD206 expression levels after addition of glutamate into microglia cultures (Figure 5D). These results suggest that although GAC overexpression is sufficient to induce microglial activation, the activation is independent of autocrine secretion of glutamate.

### GAC Overexpression Accelerates Exosome Release From Microglia

**FIGURE 6:**
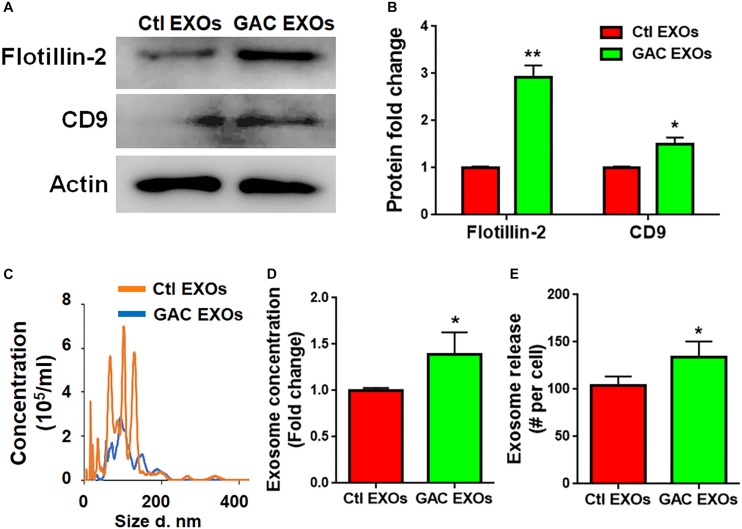
GAC overexpression increases exosome release. Exosomes released from microglia with and without GAC overexpression were collected through gradient ultracentrifugation. **(A,B)** Representative blot and quantification of CD-9 and Flotillin-2 protein expressions. **(C–E)** Size and concentration of exosomes released from microglia with and without GAC overexpression were isolated from culture supernatants and visualized through NTA **(C)**. Quantification of exosome concentration **(D)** and number of exosomes released per cell **(E)** were performed through NTA. Error bars denote s.d. from triplicate measurements. ^*^P < 0.05, ^∗∗^*P* < 0.01, by two-tailed *t*-test (*n* = 3). Experiments were carried out three times in triplicates with 5–7 P1 mice per group for *in vitro* experiments.

To determine the mechanism(s) of GAC-mediated microglial activation, we investigated exosome secretion by microglia after GAC overexpression. Our previous studies revealed that GLS1 regulated exosome release in HIV-1-infected macrophages and BV2 microglia cell lines (Wu et al., 2018). To investigate whether GAC had similar regulatory role on exosome release of primary microglia, we examined the concentrations of exosomes released from equal number of microglia with or without GAC overexpression. First we validated exosomes isolated from GAC-overexpressed microglia and control microglia by examining the expression of exosome markers CD-9 and Flotillin-2 (Figure 6A). The protein lysate from both groups generated specific bands for CD-9 and Flotillin-2, confirming the successful isolation of exosomes through ultra-centrifugation. Quantitative analyses of CD-9 and Flotillin-2 revealed higher levels of CD-9 and Flotillin-2 in GAC overexpression group, compared to control, suggesting a positive effect of GAC on microglia exosome release (Figure 6B). Consistent with the data on exosome markers, NTA revealed that although the exosome sizes were similar between GAC overexpression and control groups (Figure 6C), exosome concentrations were significantly higher in GAC overexpression group compared to the control group (Figure 6D). Calculation on the number of exosomes released per cell through dividing the number of exosomes by the number of plated microglia revealed that control microglia released about 100 exosomes while microglia with GAC overexpression released 120 exosomes in 48 h (Figure 6E). We assumed that numbers of microglia in the culture did not change since no significant difference was observed when microglia were transfected with control or GAC overexpression plasmid at 0 h and 48 h (Supplementary Figure S4). Together, our results demonstrate that GAC overexpression increases exosome release from primary microglia.

### GAC Overexpression Alters the Function and Content of Microglia-Derived Exosomes

**FIGURE 7:**
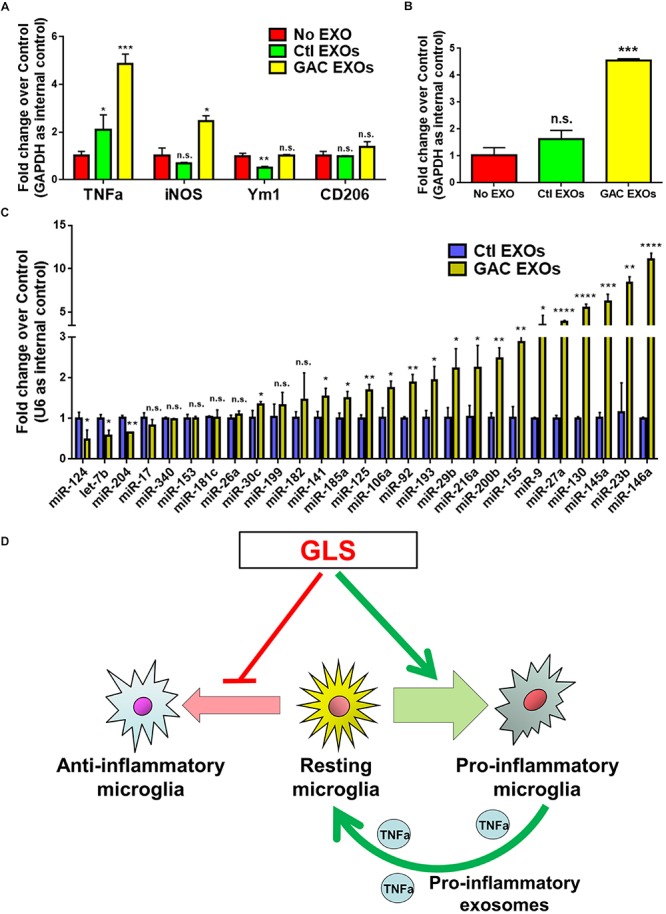
Functional and content analyses of exosomes released from GAC-activated microglia. **(A,B)** Primary mouse microglia were treated with exosomes released from GAC-overexpressed microglia or control microglia. Total RNA was collected from microglia after co-cultured with exosomes for 24 h. mRNA levels of *TNF-α, iNOS*, *Ym1*, and *CD206* **(A)** and *GAC* **(B)** were determined by qPCR. Data were normalized to *GAPDH* and presented as fold change compared to control microglia. Error bars denote s.d. from triplicate measurements. **(C)** Levels of pro-inflammatory miRNAs and anti-inflammatory miRNAs in exosomes were determined from control microglia and GAC-activated microglia through qPCR analyses. Data were normalized to U6 snRNA and presented as fold change compared to exosomes from control microglia. Error bars denote s.d. from triplicate measurements. ^*^P < 0.05, ^∗∗^*P* < 0.01, ^∗∗∗^*P* < 0.001, ^****^P < 0.0001, by two-tailed *t*-test (*n* = 3). Experiments were carried out three times in triplicates with 5–7 P1 mice per group. **(D)** A schematic representation of GAC-induced microglial activation.

To study the functional impacts of exosomes derived from GAC-overexpressed microglia, we added exosomes (15 μg exosome protein/mL medium) derived from control or GAC-overexpressed microglia to mouse microglia for 24 h. qPCR analyses revealed significant increase of transcripts corresponding to pro-inflammatory molecules, including *TNF-α* and *iNOS*, indicating that exosomes from GAC-activated microglia are sufficient to induce the inflammatory phenotype in quiescent/resting microglia (Figure 7A). Interestingly, *GAC* mRNA levels were substantially increased in microglia treated with exosomes derived from GAC-activated microglia (Figure 7B), which indicates a positive feedback loop of GAC transcription through exosomes. Because exosome mainly functions through small non-coding RNAs, we investigated the miRNA content and found significant upregulations of classic pro-inflammatory miRNAs (such as *miR-130*, *miR-145a*, *miR-23b*, and *miR-146a*, etc.) and downregulations of anti-inflammatory miRNAs (such as *miR-124* and *let-7b*) in exosomes from GAC-activated microglia (Figure 7C). These concerted alterations of exosome content suggest that activation of microglia by GAC overexpression is closely associated with microglia-derived exosomes that favor pro-inflammatory microenvironment (Figure 7D).

## Discussion

Brain inflammation, neuronal, and synaptic injury are key pathological features of AD (Sardi et al., 2011). The degree of these pathological features is often in line with the severity of AD (Kashani et al., 2008). Microglia are the residential macrophages of the CNS, regulating brain inflammation as well as neural and synaptic activity in defense against pathogens, wounds, and injuries (Block and Hong, 2005; Glass et al., 2010). Recent studies support a central role of microglia that not only instigates immune activation but also sustains elevated expression and release of pro-inflammatory mediators for AD etiopathology (Tan et al., 1999; Sastre et al., 2006). In this paradigm, investigations on regulators and mechanism(s) of microglial activation show great promise to unmask causes for AD and identify potential therapeutic targets for early AD interventions.

In the current study, we investigated whether GAC alteration had a causal effect on microglial activation. We demonstrated a significantly heightened expression of GAC in early stages of AD mouse brain tissues (Figure 1) and in mouse microglia after pro-inflammatory activation (Figure 2). Overexpression of GAC, but not KGA, led to microglial activation and inflammation (Figure 3). Importantly, glutaminase inhibitor BPTES was able to abolish LPS-induced microglial activation (Figure 4). Furthermore, GAC overexpression induced a large increase in exosome release (Figure 5), and exosomes released from GAC-overexpressed microglia activated resting microglia, which is associated with drastic upregulations of pro-inflammatory miRNAs and downregulations of anti-inflammatory miRNA in exosomes (Figure 6). Together, these results demonstrate a causal effect of GAC overexpression on microglial activation and inflammatory exosome release that are relevant to early AD pathogenesis.

The causal effect of heightened GAC expression on microglial activation has important clinical implications. One of the characteristics of AD is that there is often a long lag (10 to 20 years) for the first sign of symptoms (memory decline, disorientation, motivation loss, etc.) to occur in patients after the onset of pathological alterations in the CNS. This lag may serve as a promising therapeutic window that could be researched for novel approaches to stop or at least slow down AD progress. In the clinics, the majority of AD patients are diagnosed after symptoms occur. Therefore, therapeutic interventions often have little effects to alleviate clinical symptoms. Through current investigations, we found elevated levels of GAC expressions specifically in the 3 and 6 months APP/PS1 mouse brains. With the progression of AD, GAC levels halted their increases in 9 months APP/PS1 mouse brain, likely due to neural damage and loss in the later stages of AD. The elevation of GAC expression closely associated with the temporal pattern of plaque formation, which, starts to occur around at 3 months in mice (López-González et al., 2015). The correlation indicates the strong association of GAC deregulation and neuroinflammation with the appearance of classical AD pathological attributes such as Aβ plaque formation. Thus, our results suggest that GAC expression changes occur very early and could implicate pathological progression of AD.

It is interesting to note that only GAC, but not KGA (the other splice variant of GLS1), is significantly heightened in both early stage AD mouse brain tissues and LPS-induced pro-inflammatory microglia (Figures 1, 2). These data are consistent with the specific upregulation of GAC in HIV-infected microglia in our previous finding (Huang et al., 2011). GAC and KGA share the same DNA sequence on mitochondrial localization signal and core catalytic domain, but possess unique C-terminals due to alternative splicing at post-transcriptional modification of mRNAs (Elgadi et al., 1999; Aledo et al., 2000; de la Rosa et al., 2009). Cellular location of KGA and GAC differ in cancer cells (Cassago et al., 2012). Literatures suggest that because of the shortened 3′ UTR of GAC mRNA, miRNAs capable of targeting KGA mRNA, such as *miR-23*, are unable to bind to GAC mRNA (Gao et al., 2009; Xia et al., 2014; Masamha et al., 2016; Liu et al., 2018). In our study, we observed a dynamic complimentary upregulation of the above miRNAs in LPS-activated microglia to reverse the ongoing and toxic upregulation of KGA and GAC (data not shown). Thus, elevated KGA mRNAs are likely targeted by miRNAs, but GAC mRNAs may be more difficult to be targeted compared to KGA.

GAC is one of the splice variants of GLS1. In the CNS, GLS1 expression mainly follows a neuron-specific pattern under physiological circumstance (Ye et al., 2013). Normally, GLS1 activity and expression are mainly found in neuron-rich regions such as cortex but at lower levels in myelin-rich areas (Najlerahim et al., 1990; Botman et al., 2014). However, GLS1 activity and expression are also detected in astrocytes with lower levels than that in neurons (Kvamme et al., 2001). As the rate-limiting enzyme for glutaminolysis, GLS1 asserts control over the metabolic conditions of CNS cells. Our previous studies revealed the involvement of GLS1 deregulation in neuroinflammation and toxic effect of various cultured CNS cells, including macrophages, microglia (Zhao et al., 2004; Huang et al., 2011; Tian et al., 2012), and neurons (Ye et al., 2013; Hoffman et al., 2016). Moreover, GLS1 deregulation has been implicated in various neurodegenerative and neuropsychiatric disorders (Werner et al., 2001; Gluck et al., 2002; D’Alessandro et al., 2011; Huang et al., 2011; Zhao et al., 2012; Burbaeva et al., 2014). The current study demonstrates a causal role of heightened GAC expression on microglial activation (Figure 3), shedding new insights into neuroinflammation mechanisms along with our previous finding that mice with GAC overexpression in the CNS exhibited neuroinflammation and memory deficits (Wang et al., 2017). GAC as a mitochondrial enzyme has a key role in cellular bioenergetics and metabolism. Cellular metabolism has recently gained increasing attention as mediators of inflammatory responses of immune cells as indicated by the shifting of resting microglia and macrophages to pro-inflammatory states through glutamine synthetase inhibition (Palmieri et al., 2017a, b). Findings of the current study are in accordance with these previous reports that GAC overexpression led to microglial activation to the pro-inflammatory state. Interestingly, our results showed that GAC overexpression-induced microglial activation is not due to an increased in extracellular glutamate as evidenced by the data showing that addition of glutamate to culture media caused neither microglial activation (Figure 5C) nor apoptosis (Supplementary Figure S5). Microglia did not express inotropic glutamate receptors and metabotropic glutamate receptors except mGluR2/3 (data not shown). These data indicated that GAC-induced microglial activation is in a glutamate-independent mechanism, which is likely to be closely associated with changes in cellular metabolism. Unlike GAC, we did not observe any effects of KGA on the inflammatory responses of microglia *in vitro*. One possible explanation is that KGA may be less involved in cellular bioenergetics and metabolism due to its cytoplasmic localization, reported in cancer cells, which needs to be further clarified in microglia (Cassago et al., 2012).

Our previous studies demonstrate an increase in exosome release from activated macrophages (Wu et al., 2018). The current study revealed that GAC overexpression promoted exosome release from primary microglia. Furthermore, a substantially altered pro-inflammatory (as evidenced by increase in pro-inflammatory miRNAs and decrease in anti-inflammatory miRNAs) miRNA expression profile was observed in exosomes released from GAC overexpressed microglia (Figure 7), indicating that exosomal miRNAs from AD patients’ cerebral spinal fluid or plasma might be promising candidates of biomarkers for early AD diagnosis.

In summary, the current study revealed a heightened expression of GAC in early stage AD mouse brain tissues and pro-inflammatory mouse primary microglia. This heightened expression is sufficient to induce microglial activation and make functional changes to exosomes and exosome content in microglia. These results strongly implicate the role of GAC-mediated gene expression and exosome release on microglial activation in the early pathogenesis of AD.

## Data Availability

The raw data supporting the conclusions of this manuscript will be made available by the authors, without undue reservation, to any qualified researcher.

## Ethics Statement

All work involving animals was approved by the Institutional Animal Care and Use Committee of the Tongji University School of Medicine.

## Author Contributions

XX, YW, YH, and JCZ conceived and designed the experiments. GG, SZ, XX, CHL, CCL, CJ, YT, SS, and JZ performed the experiments. GG, SZ, XX, and YW analyzed the data. GG, SZ, XX, YW, YH, and JCZ contributed to the reagents, materials, and analysis tools. XX, YW, YH, and JCZ wrote the manuscript.

## Conflict of Interest Statement

The authors declare that the research was conducted in the absence of any commercial or financial relationships that could be construed as a potential conflict of interest.

## Footnotes

1 http://rsb.info.nih.gov/nih-image/

## Abbreviations

AD: Alzheimer’s disease
CNS: central nervous system
GAC: glutaminase C
GLS1: glutaminase 1
KGA: kidney-type glutaminase
LPS: lipopolysaccharides
NTA: nanoparticle tracking analysis.
