# Supplementary material for: Glutaminase C Regulates Microglial Activation and Pro-inflammatory Exosome Release: Relevance to the Pathogenesis of Alzheimer’s Disease
Source: Front Cell Neurosci. 2019 Jun 28;13:264. doi: 10.3389/fncel.2019.00264 (PMC6611423; doi:10.3389/fncel.2019.00264)
Supplement: Supplementary file 1 [file Data_Sheet_1.docx]

**Glutaminase C regulates the activation of microglia and the release of pro-inflammatory exosomes**

Ge Gao^1ǂ^, Shu Zhao^1ǂ^, Xiaohuan Xia^1ǂ^, Chunhong Li^1^, Congcong Li^1^, Chenghui Ji^1^, Shiyang Sheng^1^, Yalin Tang^1^, Jie Zhu^1^, Yi Wang^1*^, Yunlong Huang^1,3*^, Jialin C. Zheng^1,2,3,4*^

**Supplemental Materials**

Supplemental Figure 1

Supplemental Figure 2

Supplemental Figure 3

Supplemental Figure 4

Supplemental Figure 5

Supplemental Table 1

**
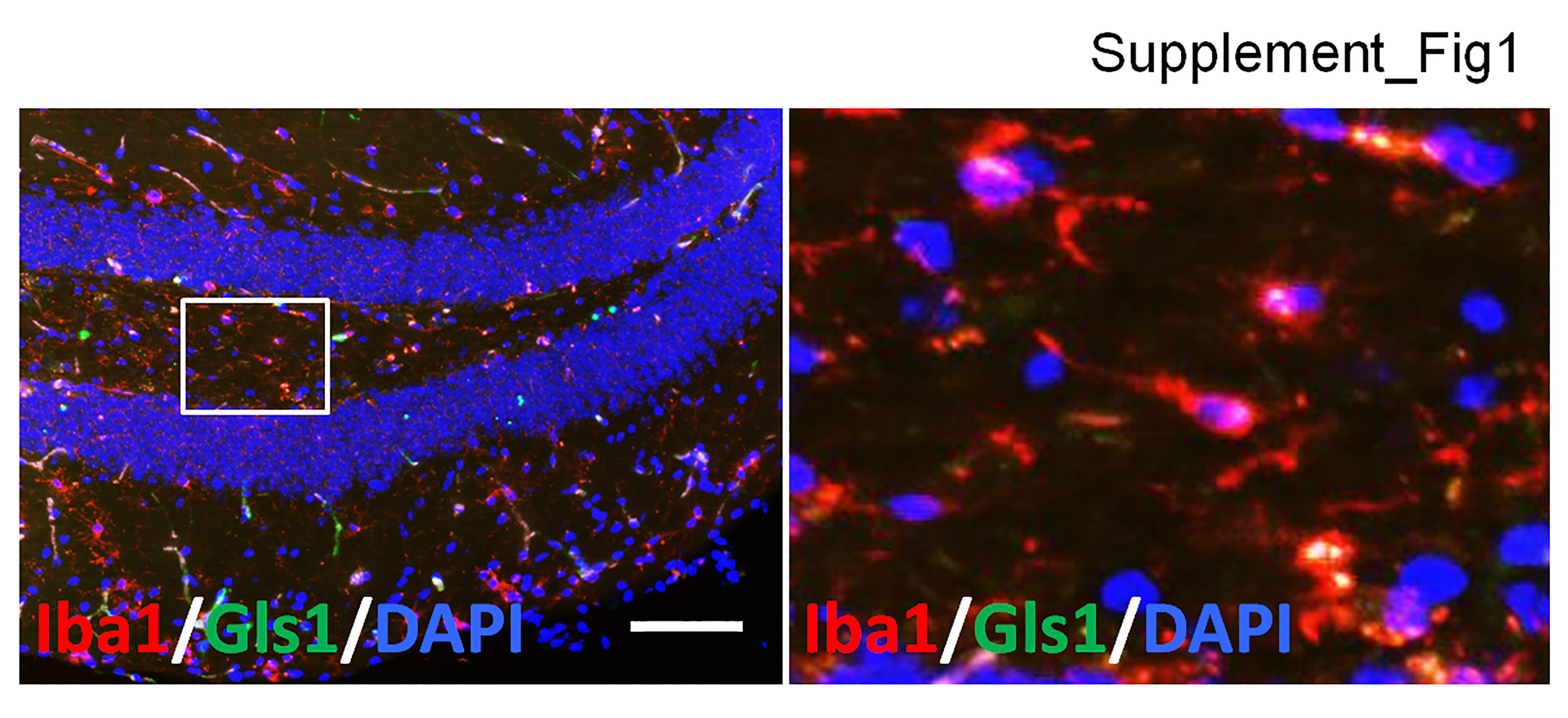
**

**Supplemental Figure 1. The co-expression of Gls1 and Iba1 in the AD mouse hippocampus at 18 months old.** The expressions of Gls1 and Iba1 in the AD mouse hippocampus at 18 months old were visualized by Gls1 and Iba1 immunofluorescent co-staining. Representative pictures are shown. The white box in the left panel was magnified in the right panel. Scale bar: 100 μm.

**
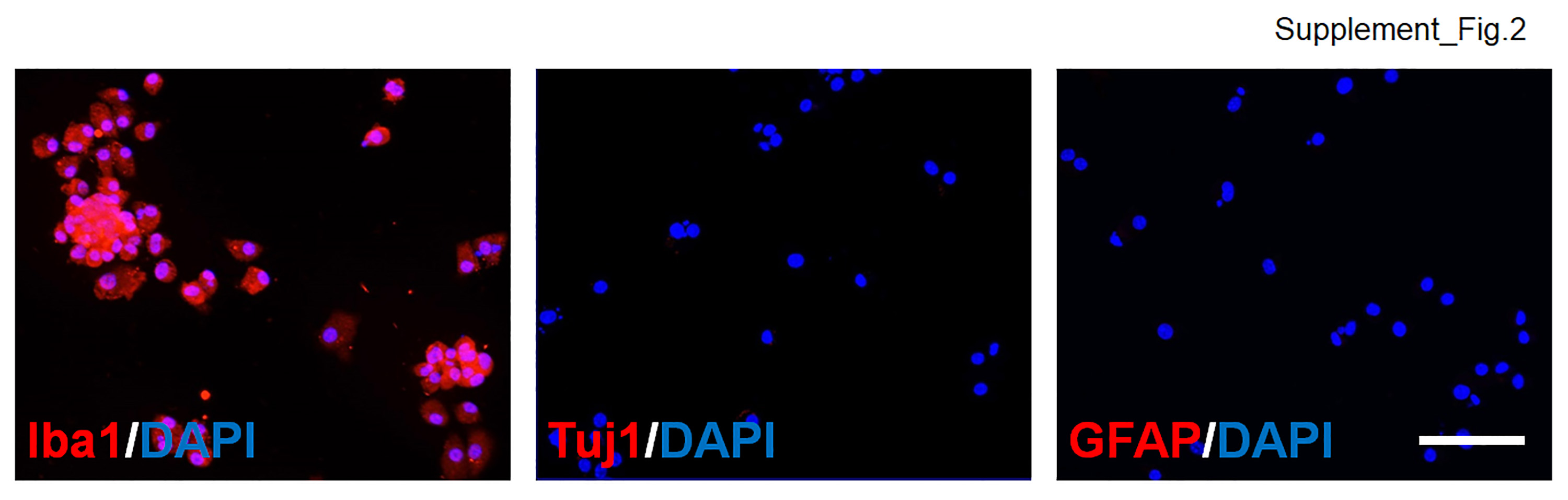
**

**Supplemental Figure 2. The validation of microglia enrichment *in vitro.*** Mouse primary microglia were counterstained with Iba-1 (microglia marker), Tuj1 (neuron marker), and GFAP (astrocyte marker) for the validation of the purity of microglia culture. Scale bar = 50 μm

**
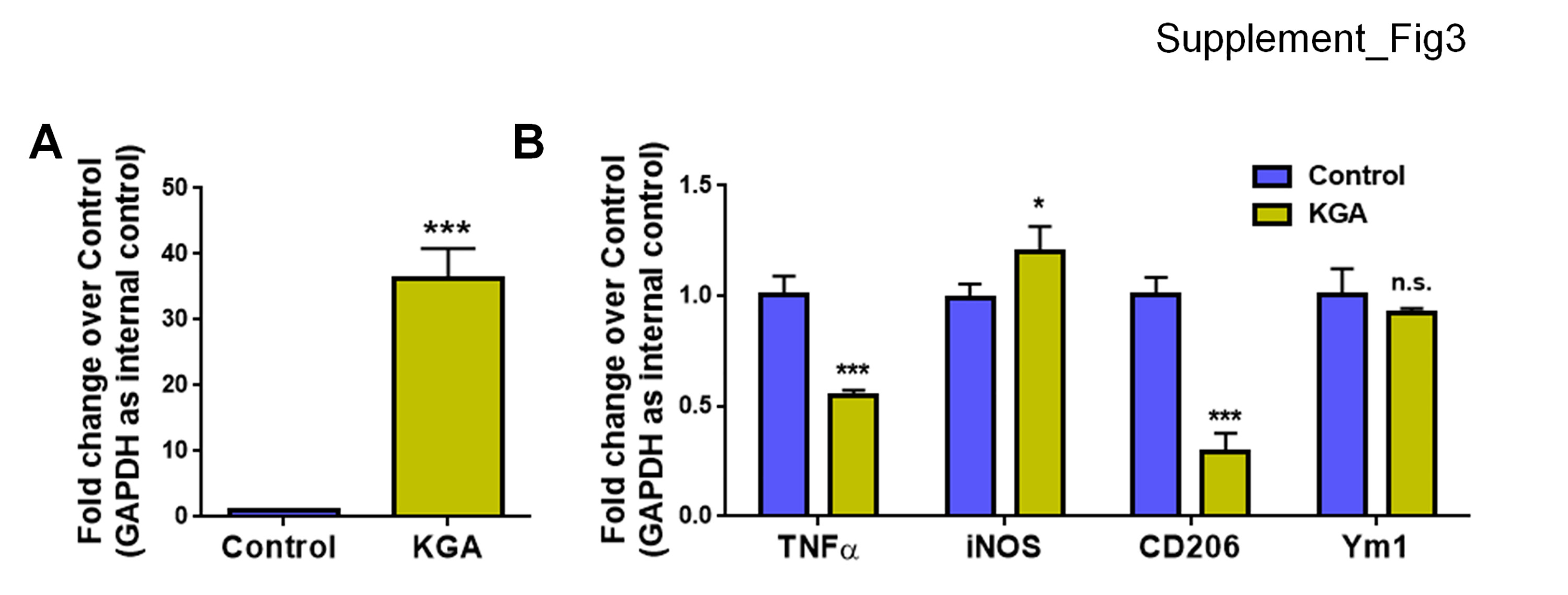
Supplemental Figure 3. The overexpression of KGA did not affect the activation of microglia *in vitro*.** (A-B) Primary mouse microglia were transfected with KGA overexpressing plasmid for 48 hours. Total RNA was collected. (A) mRNA levels of *KGA*. Data were normalized to GAPDH and presented as fold change compared to control microglia. Error bars denote s.d. from triplicate measurements. (B) mRNA levels of *TNF-α*, *iNOS*, *CD206* and *Ym1*. Data were normalized to GAPDH and presented as fold change compared to control microglia. Error bars denote s.d. from triplicate measurements. *P < 0.05, ***P < 0.001, by two-tailed t test (n = 3). Experiments were carried out three times in triplicates with 5 - 7 P1 mice per group for *in vitro* perturbation.

**
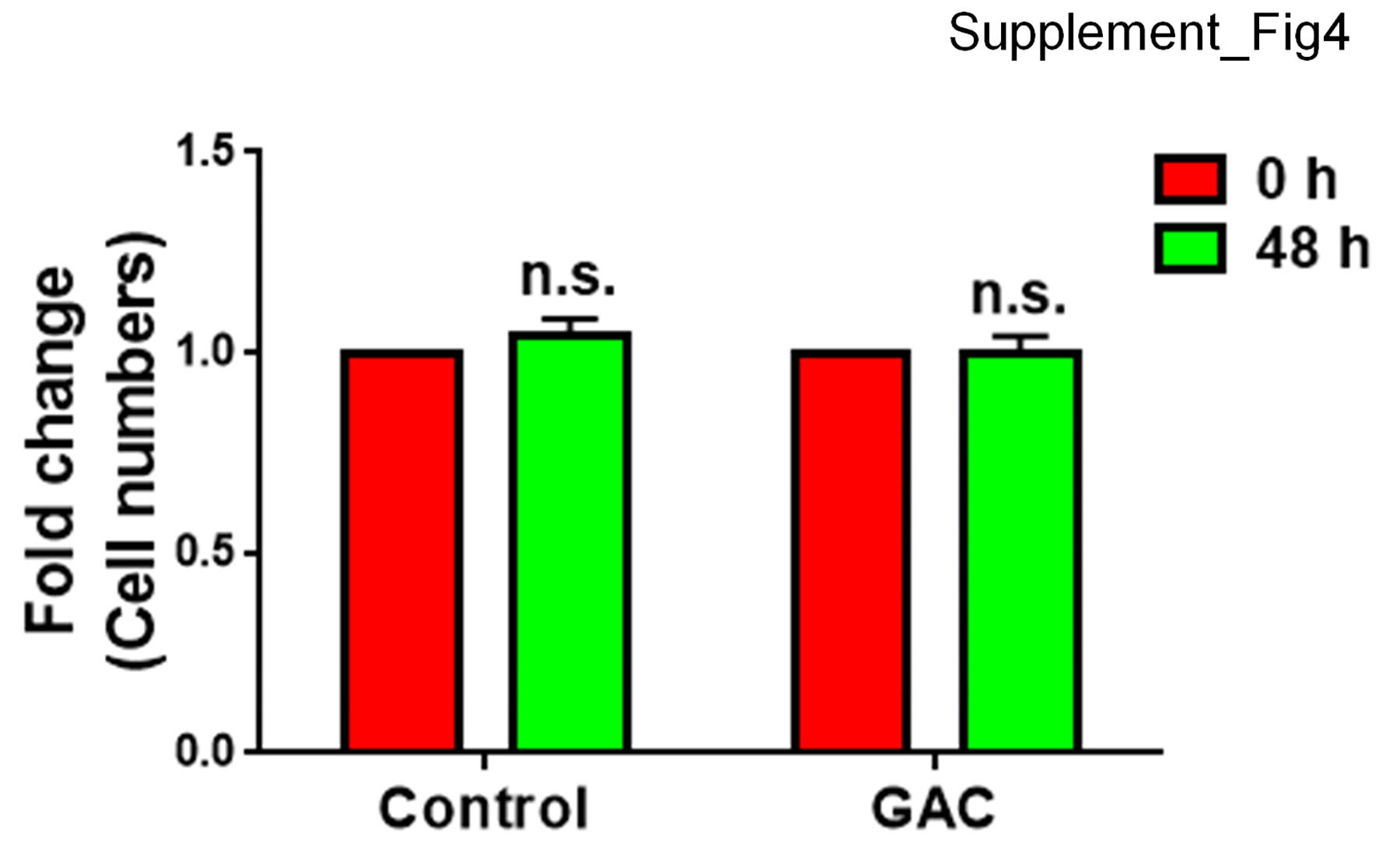
**

**Supplemental Figure 4. The plasmid transfection did not affect the number of microglia *in vitro*.** The microglia numbers at 0 h and 48 h post transfection were quantified and fold changes were made for both control and GAC overexpression conditions. Error bars denote s.d. from triplicate measurements. Experiments were carried out three times in triplicates *in vitro* perturbation.

**
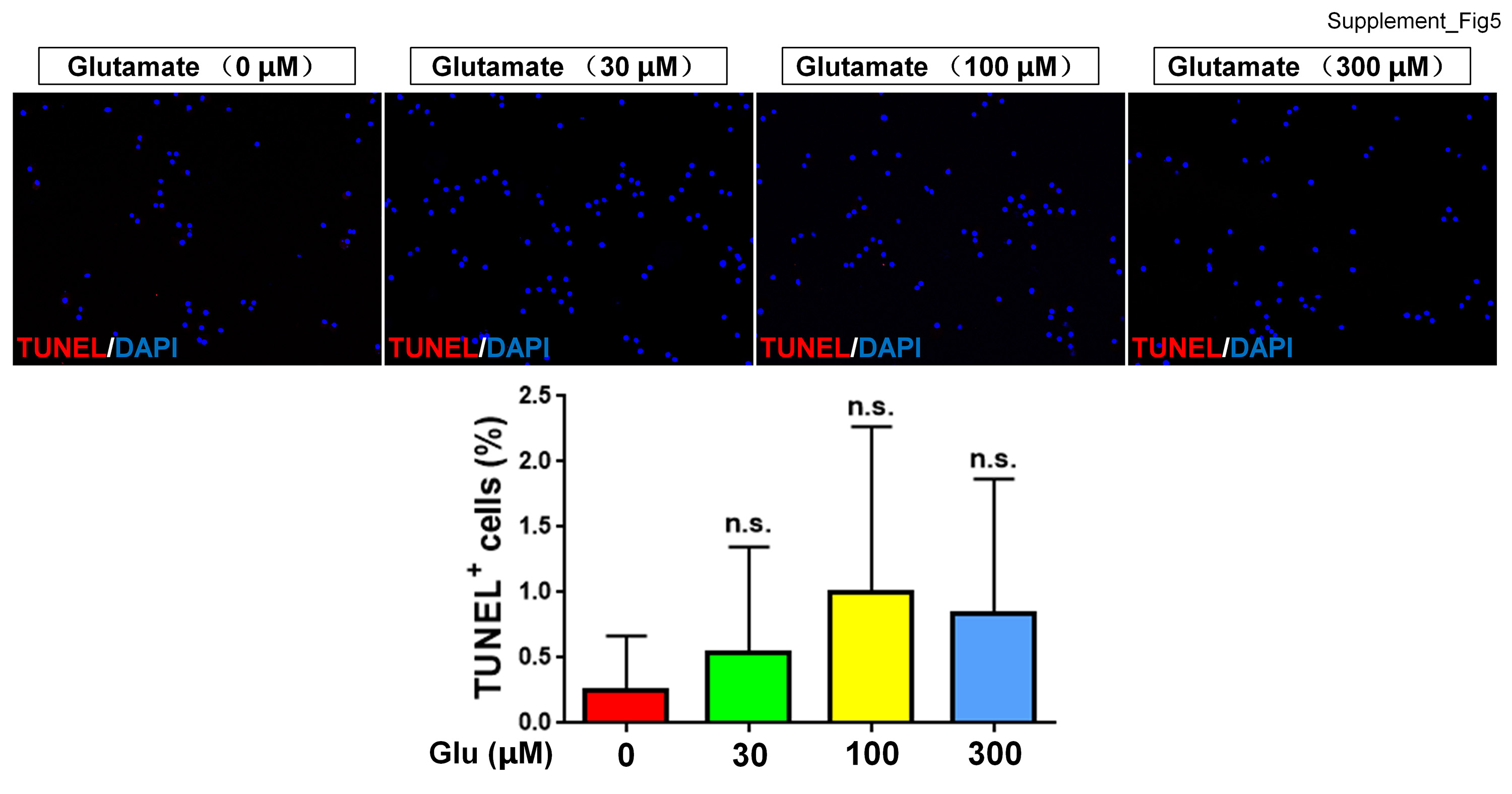
**

**Supplemental Figure 5. The treatment of glutamate did not induce microglia apoptosis *in vitro*.** Microglia apoptosis was determined by terminal deoxynucleotidyl transferase dUTP nick end labeling (TUNEL) assay. The percentage of TUNEL+ cells was determined by counting the number of TUNEL+ cells over the number of DAPI+ cells in each microscope field. Error bars denote s.d. of 10 fields in each experimental group. Experiments were carried out three times in triplicates.

**Supplement Table 1. List of specific primers.**

| Gene | Sequence | Size(bp) | T^o^ | Accession N. |
| --- | --- | --- | --- | --- |
| *Arg1* | 5’-TTTTAGGGTTACGGCCGGTG -3’  5’-CCTCGAGGCTGTCCTTTTGA-3’ | 146 | 57 | NM_007482.3 |
| *CD206* | 5’-TCTTTGCCTTTCCCAGTCTCC-3’  5’-TGACACCCAGCGGAATTTC-3’ | 241 | 56 | NM_008625.2 |
| *GAPDH* | 5’-CATGTTCCAGTATGACTCCACTC-3’  5’-GGCCTCACCCCATTTGATGT-3’ | 136 | 60 | NM_001289726.1 |
| *GLS1 (KGA)* | 5’-TTGTCCCCAACGTCATGGGC-3’  5’-TCTGCTGCTGCGACATGGAG-3’ | 254 | 57 | NM_001081081.2 |
| *GLS1 (GAC)* | 5’-TTGTCCCCAACGTCATGGGC-3’  5’-GGAGGGCAGACACATCTCCA-3’ | 237 | 57 | NM_001081081.2 |
| *iNOS* | 5’-CCCTTCAATGGTTGGTACATGG-3’  5’-ACATTGATCTCCGTGACAGCC-3’ | 158 | 57 | NM_001313922.1 |
| *TNFa* | 5’-ACGTGGAACTGGCAGAAGAG-3’  5’-GGTCTGGGCCATAGAACTGA-3’ | 206 | 57 | NM_013693.3 |
| *Ym1* | 5’-TCACAGGTCTGGCAATTCTTCTG-3’  5’-ACTCCCTTCTATTGGCCTGTCC-3’ | 101 | 57 | NM_009892.3 |
| miRNA |  |  |  |  |
| Universal primer | 5’-GAATCGAGCACCAGTTACGC-3’ |  |  |  |
| *U6* | 5’-TGGCCCCTGCGCAAGGATG-3’ |  | 55 |  |
| *let-7b* | 5’-TGAGGTAGTAGGTTGTGTGGTT-3’ |  | 55 | MIMAT0000522 |
| *miR-106a* | *5’-*CAAAGTGCTAACAGTGCAGGTAG*-3’* |  | 55 | MIMAT0000385 |
| *miR-124a* | *5’-*TAAGGCACGCGGTGAATGCC*-3’* |  | 55 | MIMAT0000716 |
| *miR-125a* | *5’-* TCCCTGAGACCCTTTAACCTGTGA *-3’* |  | 55 | MIMAT0000135 |
| *miR-130a-3p* | *5’-*CAGTGCAATGTTAAAAGGGCAT*-3’* |  | 55 | MIMAT0000141 |
| *miR-141* | *5’-*TAACACTGTCTGGTAAAGATGG*-3’* |  | 55 | MIMAT0000153 |
| *miR-145a-5p* | *5’-*GTCCAGTTTTCCCAGGAATCCCT*-3’* |  | 55 | MIMAT0000157 |
| *miR-146a-5p* | *5’-*TGAGAACTGAATTCCATGGGTT*-3’* |  | 55 | MIMAT0000158 |
| *miR-153-3p* | *5’-*TTGCATAGTCACAAAAGTGATC*-3’* |  | 55 | MIMAT0000163 |
| *miR-155* | *5’-*TTAATGCTAATTGTGATAGGGGT*-3’* |  | 55 | MIMAT0000165 |
| *miR-17* | *5’-*CAAAGTGCTTACAGTGCAGGTAG*-3’* |  | 55 | MIMAT0000386 |
| *miR-181c* | *5’-*AACATTCAACCTGTCGGTGAGT*-3’* |  | 55 | MIMAT0000674 |
| *miR-185-5p* | *5’-TGGAGAGAAAGGCAGTTCCTGA-3’* |  | 55 |  |
| *miR-19a-3p* | *5’-TGTGCAAATCTATGCAAAACTGA-3’* |  | 55 | MIMAT0000651 |
| *miR-193a-3p* | *5’-AACTGGCCTACAAAGTCCCAGT-3’* |  | 55 | MIMAT0000223 |
| *miR-199a-5p* | *5’-CCCAGTGTTCAGACTACCTGTTC-3’* |  | 55 | MIMAT0000229 |
| *miR-200b-3p* | *5’-*TAATACTGCCTGGTAATGATGA*-3’* |  | 55 | MIMAT0000233 |
| *miR-204-5p* | *5’-*TTCCCTTTGTCATCCTATGCCT*-3’* |  | 55 | MIMAT0000237 |
| *miR-216a-5p* | *5’-*TAATCTCAGCTGGCAACTGTGA*-3’* |  | 55 | MIMAT0000662 |
| *miR-23b* | *5’-*ATCACATTGCCAGGGATTACC*-3’* |  | 55 | MIMAT0000125 |
| *miR-27b-3p* | *5’-*TTCACAGTGGCTAAGTTCCGC*-3’* |  | 55 | MIMAT0000537 |
| *miR-29b-3p* | *5’-*TAGCACCATTTGAAATCAGTGTT*-3’* |  | 55 | MIMAT0000127 |
| *miR-30c-1-3p* | *5’-*CTGGGAGAGGGTTGTTTACTCC*-3’* |  | 55 | MIMAT0000416 |
| *miR-340-5p* | *5’-*TTATAAAGCAATGAGACTGATT*-3’* |  | 55 | MIMAT0004651 |
| *miR-9* | *5’-*TCTTTGGTTATCTAGCTGTATGA*-3’* |  | 55 | MIMAT0000142 |
| *miR-92* | *5’-*TATTGCACTTGTCCCGGCCTG*-3’* |  | 55 | MIMAT0000539 |
